# Signaling Pathways of Type I and Type III Interferons and Targeted Therapies in Systemic Lupus Erythematosus

**DOI:** 10.3390/cells8090963

**Published:** 2019-08-23

**Authors:** I-Tsu Chyuan, Hong-Tai Tzeng, Ji-Yih Chen

**Affiliations:** 1 Department of Internal Medicine, Cathay General Hospital, Taipei 10630, Taiwan; 2 Department of Medical Research, Cathay General Hospital, Taipei 10630, Taiwan; 3 School of Medicine, College of Medicine, Fu Jen Catholic University, New Taipei City 24205, Taiwan; 4 Institute for translational research in biomedicine, Kaohsiung Chang Gung Memorial Hospital, Kaohsiung 83301, Taiwan; 5 Department of Medicine, Division of Allergy, Immunology and Rheumatology, Chang Gung Memorial Hospital, Taoyuan 33375, Taiwan; 6 College of Medicine, Chang Gung University, Taoyuan 33375, Taiwan

**Keywords:** interferon, interferon receptor signaling, dendritic cell, T cell, systemic lupus erythematosus

## Abstract

Type I and type III interferons (IFNs) share several properties in common, including the induction of signaling pathways, the activation of gene transcripts, and immune responses, against viral infection. Recent advances in the understanding of the molecular basis of innate and adaptive immunity have led to the re-examination of the role of these IFNs in autoimmune diseases. To date, a variety of IFN-regulated genes, termed IFN signature genes, have been identified. The expressions of these genes significantly increase in systemic lupus erythematosus (SLE), highlighting the role of type I and type III IFNs in the pathogenesis of SLE. In this review, we first discussed the signaling pathways and the immunoregulatory roles of type I and type III IFNs. Next, we discussed the roles of these IFNs in the pathogenesis of autoimmune diseases, including SLE. In SLE, IFN-stimulated genes induced by IFN signaling contribute to a positive feedback loop of autoimmunity, resulting in perpetual autoimmune inflammation. Based on this, we discussed the use of several specific IFN blocking strategies using anti-IFN-α antibodies, anti-IFN-α receptor antibodies, and IFN-α-kinoid or downstream small molecules, which intervene in Janus kinase (JAK)-signal transducer and activator of transcription (STAT) pathways, in clinical trials for SLE patients. Hopefully, the development of novel regimens targeting IFN signaling pathways will shed light on promising future therapeutic applications for SLE patients.

## 1. Introduction

The systemic lupus erythematosus (SLE) is characterized by a wide array of immune tolerance breakdown with systemic inflammation involving the dysregulation of immune responses. Due to the complexity of its clinical manifestations, limitations of laboratory examination, and a current lack of effective medication, SLE is one of the most difficult-to-control autoimmune diseases. The incidence of SLE varies around the world, with the highest incidence reported in North America (23.2/100,000 person-years) [1] and the lowest incidence reported in Africa (0.3/100,000 person-years) [2]. In general, European countries have lower incidences of SLE, while Asia, Australasia, and the Americas have higher incidences [3]. For each age and ethnic group, women are more vulnerable to SLE than men, and most cases in women occur in middle adulthood. Although the differences in epidemiology results remain unclear, the pathogenesis of SLE appears to involve a complex interplay of immunological, genetic, and environmental risk factors. Advancements in genome-wide association studies (GWAS) have at least partially elucidated the complex genetic architecture of SLE and have identified differences in risk variants across different continental populations [4,5]. More than 90 risk loci have been identified and have collectively been used to establish several critical pathways involved in SLE pathogenesis, including innate immune responses, lymphocyte activation, and immune complex clearing [6]. In particular, these pathways result from the inadequate clearance of nuclear debris and immune complexes, the dysregulation of the innate immune system driven by innate receptors, such as Toll-like receptors (TLRs) and interferon (IFN) signaling, and aberrant immune responses of the adaptive immune system mediated by B-cell and T-cell signals [7]. In serial gene expression microarray studies, the upregulation of IFN-inducible genes (IFIGs) contributes to over 50% of pathogenesis in human SLE patients [8,9], suggesting a strong association between IFNs and the development of SLE. As current treatments are usually of limited efficacy, it is critical to exploit novel targets related to SLE pathogenesis. Hopes have strongly rested on biological agents since several biological therapies have shown great efficacy in patients with other autoimmune rheumatic diseases. However, biological therapies for the treatment of SLE appear to be relatively unsuccessful, and newly developed biological agents have failed to meet their primary endpoints in large-scale clinical trials [10]. Given the key role of IFNs in the initiation and perpetuation of autoimmune responses in SLE, many efforts have been made to obtain an in-depth understanding of IFNs and the development of targeted SLE therapies through intervening in IFN signaling pathways.

In this review, we have focused on the signaling pathways of type I and type III IFNs and outlined their immune-regulatory function in the pathogenesis of SLE. We also discussed current therapies, which target the signaling pathways of type I and type III IFNs, and their results in large-scale clinical trials in SLE.

## 2. IFNs and IFN Signaling Pathways

IFNs are critical cytokines involved in the manipulation of complicated antiviral responses. More than 20 forms of IFN have been identified, and these have been further classified into three families–type I, type II, and type III–based on their distinct structures, receptor ligation, and biological activities. IFNs belong to the class II cytokine family (which also includes interleukin (IL)-10, IL-19, IL-20, IL-22, IL-24, and IL-26) and share a conserved structure comprised of six α-helices. The type II IFN family has only one member, IFN-γ, which mediates pro-inflammatory and immunomodulatory functions, unlike type I and type III IFNs. The role of type II IFNs in autoimmunity has been reviewed elsewhere [11] and has not been discussed herein.

### 2.1. Type I IFNs

**Figure 1:**
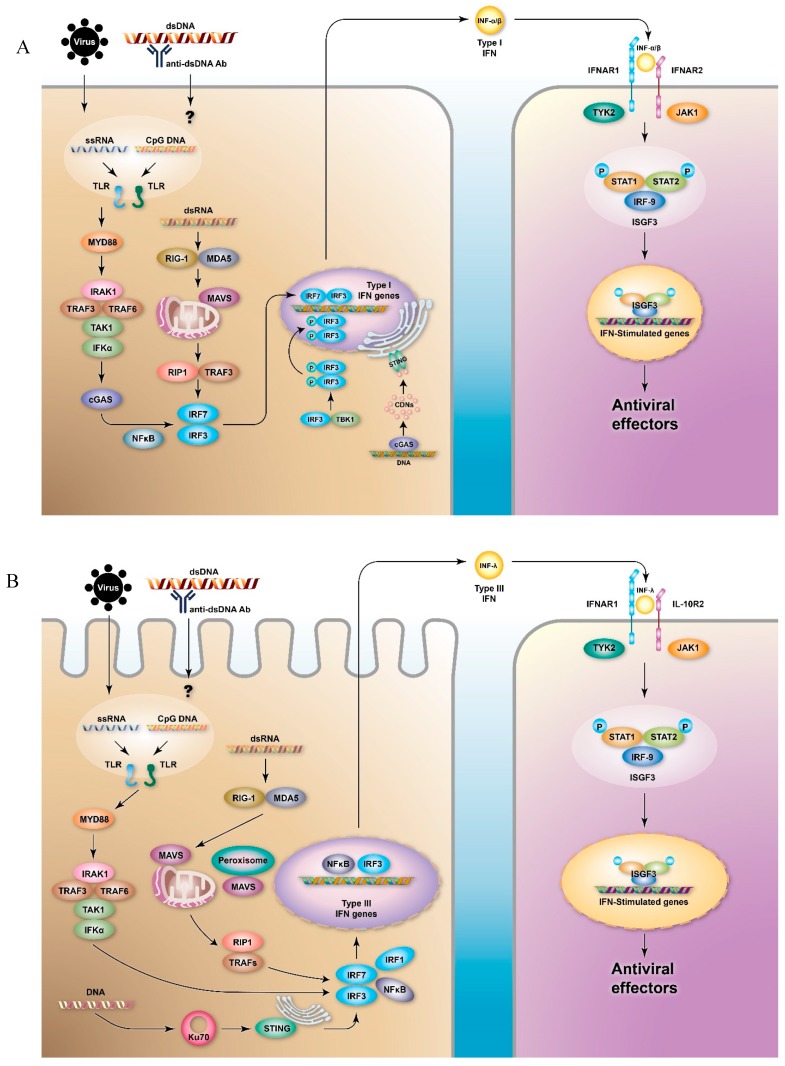
Signaling pathways of type I and type III interferons (IFNs). The production of (**A**) type I and (**B**) type III IFNs can be induced by virus infection or by immune complexes, which are sensed by pattern recognition receptors (PRRs), especially toll-like receptors (TLRs) and retinoic acid-inducible gene 1 (RIG-I)-like receptors (RLRs). Differential signaling molecules lead to the activation of the transcription factors nuclear factor kappa-light-chain-enhancer of activated B cells (NF-κB) and interferon regulatory factors (IRFs), and eventually to the activation of IFN gene transcripts. The secreted IFNs ligate to the type I or type III IFN receptors (IFN-α/β receptor (IFNAR)1/IFNAR2 or IFN-λ receptor (IFNLR)1/interleukin-10 receptor (IL-10R)2, respectively) of the neighboring cells and stimulate the production of IFN-stimulated genes (ISGs) via Janus kinase (JAK)-signal transducer and activator of transcription (STAT) pathways, which results in the production of several antiviral effectors. Cyclic GMP-AMP synthase (cGAS) transforms DNA into cyclic di-nucleotides (CDNs), which can be recognized by stimulator of interferon genes (STING). STING triggers the activation of tank-binding kinase 1 (TBK1) to phosphorylate IRF-3 and induces the transcription of type I IFNs. In the transcription of type III IFNs, the activation of STING is involved in Ku70.

Type I IFNs are the largest family of IFN and comprise five classes in humans, namely IFN-α, IFN-β [12], IFN-ω, IFN-ε, and IFN-κ [13]. Of these, the IFN-α class is the largest and can be further divided into 12 subtypes, which encode 13 highly homologous genes clustered on chromosome 9 [14]. The production of type I IFNs is induced by pathogen-associated molecular patterns (PAMPs), composed of evolutionarily conserved molecules like nucleic acids in viruses and nucleic acids, peptidoglycan, flagellin, or lipopolysaccharides (LPS) in bacteria, which are sensed by corresponding pattern recognition receptors (PRRs). PRRs include TLRs, retinoic acid-inducible gene 1 (RIG-I)-like receptors (RLRs), and nucleotide oligomerization domain (NOD)-like receptors (NLRs) [15] (Figure 1). When activated via PRRs, type I IFNs are mainly produced by plasmacytoid dendritic cells (pDCs), although type I IFNs can be produced by almost all nucleated cells [16]. Mechanistically, through the expression of TLR7 or TLR9 in endosomal membranes, pDCs sense the RNA or DNA of pathogens via receptor-mediated endocytosis and initiate an activation chain involving myeloid differentiation factor 88 (MyD88) and downstream transcription factors, such as interferon regulatory factor (IRF)-3 and IRF-7, finally inducing the expression of type I IFNs [17,18]. Additionally, type I IFN can also be produced through TLR-independent cytosolic recognition systems, which are mediated by RNA-sensing molecules. For example, cytosolic RIG-I and melanoma differentiation-associated protein 5 (MDA5) sense viral RNA and promote downstream signaling cascades involving mitochondrial antiviral-signaling protein (MAVS), tumor necrosis factor receptor-associated factor 2 (TRAF2), TRAF6, Fas-associated protein with the death domain (FADD), and receptor-interacting protein-1 (RIP1) [19,20], which eventually lead to the activation of IRF-3, IRF-7, and nuclear factor kappa-light-chain-enhancer of activated B cells (NF-kB), which in turn leads to the induction of type I IFN genes [21,22]. The production of type I IFNs is mainly controlled by various serial gene transcriptions, especially the IRF system, in a strictly highly ordered process.

Another DNA-sensing PRR is involved in the activation of stimulator of interferon genes (STING). STING is a transmembrane protein, which resides in the endoplasmic reticulum (ER) of epithelial and endothelial cells and a variety of hematopoietic cells, such as macrophages and dendritic cells (DCs). When pathogen-related DNA is present in the cytosol of a cell, cyclic GMP-AMP synthase (cGAS) transforms it into cyclic di-nucleotides (CDNs), and the resulting CDNs bind to STING. This triggers the activation of Tank-binding kinase 1 (TBK1), by which TBK1 phosphorylates IRF-3, thus inducing the transcription of type I IFNs [23] (Figure 1A). After the activation of TBK1, STING is phosphorylated by a serine/threonine UNC-51-like kinase, UNC-51-like autophagy activating kinase 1/autophagy-related protein 1 (ULK1/ATG1), and consequently, IRF-3 function is suppressed [24].

**Figure 2:**
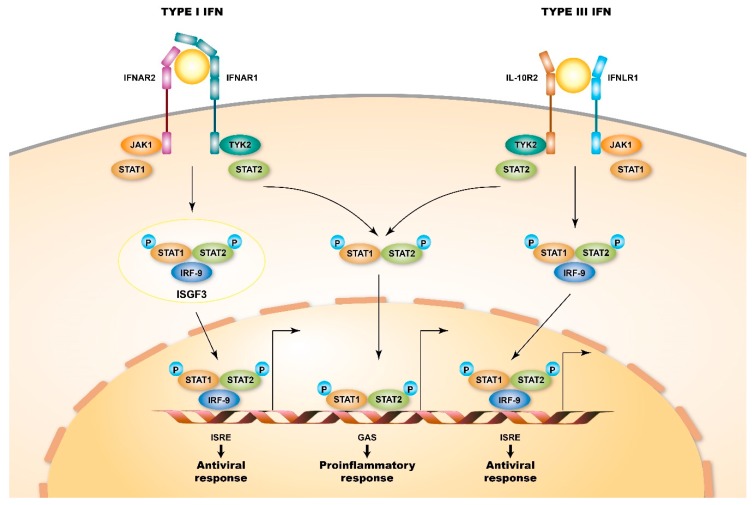
Signaling pathways of type I and type III IFN receptors. Type I and type III IFN receptors are heterodimers and consist of different receptor chains (IFNAR1 and IFNAR2 for the type I IFN receptor and IFNLR1 and IL10R2 for the type III IFN receptor). Both receptors are associated with two kinases from the JAK family: JAK1 and TYK2 (tyrosine kinase 2). When ligating to their cognate ligand, JAK kinases auto-phosphorylate the IFN receptor, which results in the recruitment of signal transducer and activator of transcription (STAT) proteins, phosphorylation, dimerization, and nuclear translocation. In particular, STAT1, STAT2, and IFN-regulatory factor 9 (IRF9) form interferon-stimulated gene factor 3 (ISGF3) complexes and bind to IFN-stimulated response element (ISRE) sequences, thus activating classical antiviral genes. Additionally, STAT1 homodimers bind to gamma-activated sequences (GASs), thus inducing the activation of pro-inflammatory genes.

All of the produced type I IFNs further exert functions via ligating to a shared heterodimeric receptor, IFN-α/β receptor (IFNAR), which is composed of IFNAR1 and IFNAR2 subunits. The expression of IFNAR is ubiquitous in most nucleated cells; however, the level of expression varies between cell types [25,26]. IFNs can especially interplay with pDC, macrophages, natural killer cells, and T- and B-cells, which increases their survival, maturation, and activity [13] and indicates their pleiotropic effects on the modulation of both the innate and the adaptive immune responses. The engagement of IFNs with IFNAR activates the receptor-associated protein tyrosine kinases Janus kinase 1 (JAK1) and tyrosine kinase 2 (TYK2), which in turn phosphorylates the latent cytoplasmic transcription factors signal transducer and activator of transcription 1 (STAT1) and STAT2. Subsequently, the phosphorylated STAT1 and STAT2 dimerize and translocate to the nucleus, and the STAT1/STAT2 dimers in the nucleus assemble with IRF-9 to form a tri-molecular complex called IFN-stimulated gene factor 3 (ISGF3). Following the activation of the transcription of interferon-stimulated genes (ISGs), ISGF3 further binds to its cognate DNA sequences, IFN-stimulated response elements (ISREs), which results in a cellular antiviral state [27,28] (Figure 1A). Additionally, type I IFN receptors can also activate phosphorylated STAT1 homodimers to bind to gamma-activated sequences (GASs), which induces the activation of pro-inflammatory genes [29] (Figure 2).

### 2.2. Type III IFNs

Type III IFNs, also called IFN-λs, are the most recently identified members of the IFN family. Type III IFNs are comprised of IFN-λ1 (IL-29), IFN-λ2 (IL-28A), IFN-λ3 (IL-28B) [30], and IFN-λ4 [31]. IFN-λs can be produced by various cell types; however, are mainly produced by pDCs [32,33], as are type I IFNs. However, intestinal epithelial cells (IECs) can preferentially produce IFN-λs [34], and the production of IFN-λs is more abundant at mucosal sites in epithelial and myeloid cells in response to viral infection [35], suggesting a predominant role of IFN-λs in the mucosal immune system response against viral infection. Similar to type I IFNs, IFN-λs are expressed following the detection of PAMPs by PRRs, such as TLRs [36] and RLRs [37]. The sensing of PAMPs by RLRs results in the recruitment of MAVS to mitochondria complemented by peroxisomes, which results in the activation of the transcription factors NF-κB and IRFs and eventually in the induction of the expression of IFN-λs [38] (Figure 1B). Importantly, the cytosolic sensor Ku70 appears to be preferentially involved in the production of IFN-λs rather than type I IFNs [39]. Although the precise mechanism remains unclear, experiments involving the transfection of DNA or herpes simplex virus-2 infection have shown that viral DNA can bind to Ku70, further recruits STING, and subsequently activates downstream IRFs [40] (Figure 1B). Furthermore, IFN-λ can be induced by type I IFNs through ISGs [41]. Besides, the type I and III IFNs that are induced following viral infection can also be temporally regulated. Type I IFNs genes are more rapidly induced and resolved; by contrast, the following *IFNL* genes are induced in a delayed but sustained fashion [42,43]. The differential expressions of type I and type III IFNs may be involved in the induction of type III IFNs and require the co-activation of both IRF and NF-κB signals [44]. Additionally, the IFN-inducible nature of IFN-λs indicates that these genes can be directly induced by ISGF3 or other IFN-induced transcription factors, such as IRF-1.

Structurally, type III IFN receptors are heterodimeric receptors, unlike type I IFN receptors, and are composed of high-affinity IFN-λ receptor (IFNLR)1 chains (IL-28Rα) and low-affinity IL-10R2 chains. Despite the difference in the structures of type I and type III IFN receptors, the engagement of IFN-λs with type III IFN receptors activates the JAK-STAT signaling cascade, recruits IRF-9, and ultimately activates ISGF3, which drives the transcription of ISGs; this is similar to the engagement of type I IFNs to their cognate type I IFN receptor. Although IFNAR is ubiquitously expressed in most cell types, the expression of IFNLR1 seems to be limited to cells with epithelial origins, such as hepatocytes and intestinal cells [45,46], and to cells with immune origins, such as natural killer (NK) cells, pDCs, and DCs [47,48,49].

## 3. Immunoregulatory Function of Type I and Type III IFNs

In addition to the primary effects on viral defense, type I IFN signaling is also involved in the activation of innate immune cells and the regulation of adaptive immune responses. Type I IFNs can promote the differentiation of monocytes into mature DCs mediated by granulocyte-macrophage colony-stimulating factor (GM-CSF) [50,51], and further induce the phenotypic maturation of DCs by enhancing the expression of major histocompatibility complex (MHC) class I, MHC class II, and co-stimulatory molecules [52,53], along with cell adhesion molecules to facilitate the migration of DCs into draining lymph nodes [54]. Additionally, IFN-α drives DCs to produce CXC-chemokine ligand 9 (CXCL9) and CXCL10, which chemoattract T cells [55]. Overall, type I IFNs enhance the ability of DCs to prime T cells in secondary lymphoid organs, which facilitates immune responses. Interestingly, when exposed to chronic viral antigens, DCs from persistently infected mice exhibit an immunotolerant phenotype characterized by increased IL-10 production and the increased expression of programmed cell death 1 ligand 1 (PD-L1), which is mediated by persistent type I IFN signaling. When the type I IFN signaling is blocked with anti-IFNAR antibodies, the immunotolerant state is reversed, DCs are further stimulated, and virus-specific CD4+ T cell immunity is enhanced, which consequently improves infection control [56,57]. Altogether, the temporal control of type I IFN signaling affects DC function; short-term IFN exposure promotes the ability of DCs to prime T-cells, while prolonged exposure can induce immune-regulatory DCs to attenuate T cell-mediated immunity against viruses.

IFNAR signaling exerts a dual effect on promoting or inhibiting T cell priming, presumably via a temporal relationship between IFNAR signaling and T cell receptor (TCR) engagement. These opposite effects are involved in different intracellular signaling pathways which are initiated after IFNAR engagement in T cells, whether concomitant with or before TCR signaling [27]. When type I IFN signaling occurs via STAT1, it leads to a pro-inflammatory, anti-proliferative, and pro-apoptotic state; however, when such signaling occurs via STAT3, STAT4, and STAT5, it leads to an anti-inflammatory, pro-proliferative, and anti-apoptotic state, which favors cell survival, proliferation, and differentiation [58,59]. The competition between STAT1 and STAT3/STAT4/STAT5 appears to result from the inability of TCR-activated T cells to phosphorylate STAT1, which leads to type I IFN signals being propagated by other STATs and consequently favors a pro-cell survival and pro-proliferative state [60].

In addition to affecting T cells, type I IFNs also induce DCs to produce B cell stimulatory cytokines, including B-cell activating factor (BAFF) and a proliferation-inducing ligand (APRIL), which cause B cells to undergo immunoglobulin class-switching [61]. Furthermore, the ability of IFN-α to augment the antibody response is greatly reduced by the conditioned deletion of IFNAR1 in B cells since B cells are unable to respond to type I IFNs; this indicates that IFN-α directly acts on B cells in the regulation of antibody responses [62]. In specific conditions, type I IFNs can also regulate the growth and survival of B-cell precursors. Direct IFN-α treatment greatly decreases bone marrow cellularity and splenic cellularity and reduces the number of B lineage cells [63], while anti-IFNAR1 antibody treatment promotes the survival and differentiation of B cells and also neutralizes the production of antibodies against lymphocytic choriomeningitis mammarenavirus (LCMV) infection [64,65]. Over-activated type I IFNs can enhance the survival of IL-6-producing transitional B cells, which has been reported to contribute to the pathogenesis of SLE [66].

Type III IFNs also modulate the responses of T- and B-cells. Studies suggest that type III IFNs can modulate T-cell responses. However, this modulation appears to be indirect and possibly occurs through DCs, since the expression of IFNLR in T cells is minimal and the response of T cells to IFN-λ remains uncertain, although some reports link IFN-λs to the skewing of T cells toward a Th1 phenotype [67]. Moreover, recent studies have shown that CD4 T-cells can express the IFN-λ1-specific receptor IL-28α, which is responsive to IFN-λ1 and further reduces existing Th2 responses by suppressing Th2 cytokines (IL-4 and IL-13) [68,69]. In addition to an increase in the number of Th1-cytokines (e.g., IFN-γ) and suppressed Th2 cytokines (e.g., IL-4, IL-5, IL-13), recombinant IL-λ3 (IL-28B) also inhibits H1N1-stimulated B cell proliferation and IgG production, and the blockade of IL-28Rα augments influenza vaccine responses [70]. In an allergic airway murine model, IL-λ2 (IL-28A) was found to ameliorate disease activity and suppress Th2 and Th17 responses with the induction of IFN-γ. Additionally, in IL-28Rα knockout mice, IL-λ2 was found to exacerbate allergic airway inflammation by augmenting Th2 and Th17 responses and IgE levels [49]. Altogether, these results suggest that IFN-λs have a Th1-skewing property and that specific virus–host and immune cell interactions possibly regulate the type III IFN signaling pathways involved in T cell proliferation and maturation. However, inconsistent results have been found across different models regarding the regulation of B cell responses by type III IFN signaling, and different IFN-λs seem to have different immunoregulatory functions. For example, IFN-λ1 can augment TLR-mediated B cell activation, possibly through the upregulation of TLR7 expression [71]. Furthermore, IFN-λ3 seems to be able to increase the production of antigen-specific anti-HIV antibodies [67]. However, conflicting results suggest that IFN-λ3 can cause an increase in IgG-production and the H1N1-stimulated activation of B cells [70], suggesting that IFN-λs can up- or downregulate B cell responses in specific triggering environments.

## 4. Type I and Type III IFNs in Autoimmunity

The dysregulated sensing of PAMPs leads to the persistent activation of innate immune signaling and eventually to autoimmune responses. IFN signaling is tightly regulated by several gene transcripts; IFN risk allele variants result in unwanted persistent IFN signaling and autoimmune inflammation, including SLE, rheumatoid arthritis (RA), and vasculitis. Various mechanisms have been proposed to cause sustained type I IFN signaling, and a resultant increase in autoimmunity has been proposed. For example, an impaired RNase H2 complex, which functions as a genome surveillance enzyme, promotes DNA strand breaks and triggers a DNA damage response [72] along with cGAS-dependent type I IFN activation [73]. Interestingly, STING knockout mice aggravate several autoimmune-disease phenotypes of lupus mouse models [74], suggesting an immune-regulatory role of STING in the pathogenesis of SLE. In humans, mutations in TMEM173, which encodes STING, have been linked to familial inflammatory syndrome with lupus-like manifestations [75]. These results suggest genetic contributions of STING to the development of SLE, probably due to aberrancy of IFN activation. Aicardi-Goutières Syndrome (AGS), a severe disease driven by type I IFNs, is characterized by progressive encephalopathy, chilblain-like skin lesions, glaucoma, hypothyroidism, and lupus-like symptoms [76]. Deficiencies in the AGS-associated genes TREX1 and SAMHD1 cause chronic low-level DNA damage and result in the activation of type I IFNs [77,78].

Genes involved in IFN signaling pathways, such as MDA5, TLR7, IRF-5, and IRF-7, are involved in the progression of Sjogren’s syndrome [79,80], a chronic autoimmune inflammatory state that involves focal lymphocytic infiltration into the exocrine glands, leading to dry eyes and mouth. Rheumatoid arthritis (RA) is autoimmune inflammatory arthritis characterized by pathological synovitis (pannus formation) and bone erosion. Genetic variants associated with the type I IFN signaling pathways have been linked with the development and progression of RA, suggesting a role of type I IFNs as a trigger factor for RA development [81]. Furthermore, the level of serum IFN-λ1 is markedly elevated in RA patients [82]. All these studies suggest that an interplay between type I and type III IFNs is involved in the pathogenesis of autoimmune diseases. Interestingly, a study of an animal model of collagen-induced arthritis (a typical mouse model for RA) revealed that treatment with IFN-λ2 reversed the development of arthritis by reducing the number of pro-inflammatory IL-17-producing Th17 and γδ T-cells in the inflamed joints and inguinal lymph nodes as well as by restricting the recruitment of IL-1β-expressing neutrophils [83]. By contrast, in the inflamed intestine of a dextran sodium sulfate (DSS)-induced colitis mouse model, IFN-λ specifically activated a translation-independent signaling pathway that diminished the production of reactive oxygen species and decreased degranulation in neutrophils [84]. Other autoimmune inflammations, such as psoriasis, are characterized by keratinocyte hyperproliferation, loss of skin barrier function, and immune cell infiltration. In psoriatic skin lesions, IFN-λ1 increases the sensitivity of keratinocytes and ISG expression, possibly by mediating Th17 cells [85]. Additionally, elevated IFN-λ expression in psoriatic lesions is associated with the upregulation of the chemokines CXCL10 and CXCL11 compared with non-lesional skin, resulting in epidermal T-cell infiltrations [86].

## 5. Type I IFNs in SLE

In 1969, it was reported that using an IFN inducer (poly I:C) accelerated autoimmune inflammation and autoantibody production in New Zealand Black and New Zealand White NZB/NZW F1hybrid strains mice, a typical animal model for the study of SLE [87]. Furthermore, in human SLE patients, observational studies have shown that increased serum levels of IFN correlate well to disease activity [88]. Additionally, it has been reported that IFN therapy can induce autoimmunity and cause SLE [89,90]. Furthermore, GWAS of SLE patients have demonstrated that key genetic variants are involved in over-activation or regulatory deficits in the innate immune responses that are closely correlated to type I IFNs, by which IRF-5 and STAT4 risk alleles additively increase the probability of developing SLE [91,92]. Additionally, risk alleles that operate in IFN pathway genes have also been implicated in the pathogenesis of lupus in GWAS [93]. Therefore, dysregulated type I interferon signaling associated with SLE is now recognized as a new family of diseases termed “interferonopathies” [94].

**Figure 3:**
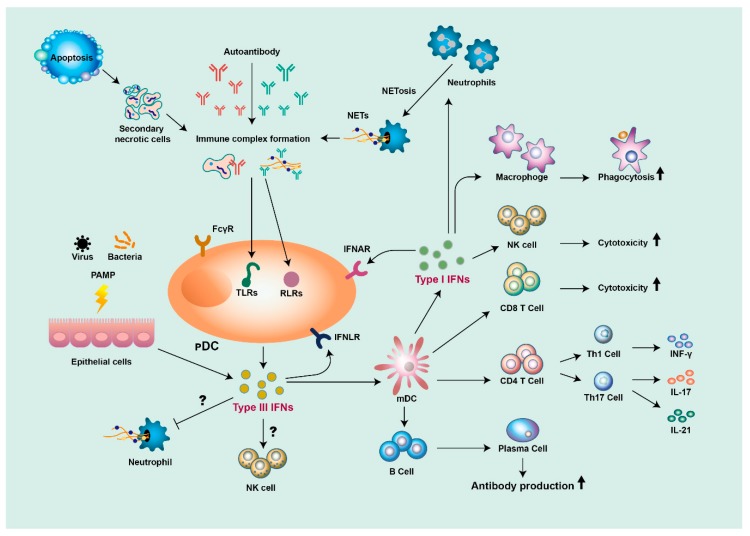
The roles of type I and type III IFNs in the pathogenesis of SLE (systemic lupus erythematosus). Self-nucleic acid from apoptotic cells, or from neutrophil extracellular traps (NETs) released from neutrophils, are detected by autoantibodies to form immune complexes, stimulating plasmacytoid dendritic cells (pDCs) to produce type I and type III IFNs. Epithelial cells also produce type III IFNs in response to pathogen-associated molecular patterns (PAMPs). Both type I and type III IFNs stimulate myeloid DCs (mDCs) to activate T- and B-cells, which leads to the production of diverse proinflammatory cytokines and autoantibodies. Type I IFNs can also promote cytotoxicity function in macrophages and natural killer (NK) cells. Both type I and type III IFNs can contribute to a positive feedback loop of inflammation.

pDCs are the main source of type I IFN production. In SLE, DNA- and RNA-containing immune complexes (ICs) are engulfed by pDCs by ligation to FcγRIIa and are then delivered into the endosomal compartment and sensed by TLR-7 and TLR-9 [95]. Another source of type I IFN production in SLE appears to be neutrophils. One of the defense mechanisms of neutrophils against pathogens is the formation of neutrophil extracellular traps (NETs), which contain primarily nuclear materials, such as dsDNA and histones. Lupus neutrophils can be primed by type I IFNs and release NETs upon exposure to SLE-derived anti-ribonucleoprotein antibodies. In turn, SLE NETs can activate pDCs, thus producing high levels of IFN-α in a TLR9-dependent fashion [96]; this suggests that the interplay between pDC and neutrophils is involved in the production of type I IFNs in SLE (Figure 3).

Although type I IFNs have been regarded as key IFNs in the pathogenesis of SLE, different classes of type I IFNs seem to have different functions in the regulation of SLE. For example, in SLE, IFN-α is a key regulator of autoimmune responses and act on multiple target cells and pathways. For instance, IFN-α stimulates the maturation of myeloid DCs from monocytes and enhances the expression of MHC class I and class II on DCs for antigen presentation [97]. Moreover, IFN-α promotes the development of CD4^+^ T-cells skewing to Th1 differentiation along with CD8^+^ T-cell maturation and suppresses the development of Th2, Th17, and regulatory T cells [98,99,100] (Figure 3). Furthermore, IFN-α stimulates the differentiation of B cells and increases the production of autoantibodies through the production of BAFF [79]. In SLE patients, IFN-α can prime monocytes for enhanced NLRP3 inflammasome activity and IL-1β production in an IRF-1-dependent manner [101], and neutrophils for NETosis upon exposure to SLE-derived anti-ribonucleoprotein antibodies [96]. 

IFN-β is mainly produced by dendritic cells, epithelial cells, and macrophages. In contrast to IFN-α, which is mainly driven by TLR activation, IFN-β is induced through the activation of the STING-IRF-3 pathway driven by the recognition of cytoplasmic nucleic acids by RNA or DNA sensors. Another difference between IFN-α and IFN-β is that IFN-β seems to play an immunoregulatory role in SLE pathogenesis; a study showed that treating patients with multiple sclerosis, an inflammatory demyelinating disease of the central nervous system, with IFN-β reduces the incidence of clinical relapses and brain disease activity [102]. The immunoregulatory function of IFN-β is possibly mediated by increased IL-10 production and the inhibition of inflammasome activation and IL-1 production [103]. Interestingly, circulating B cells from SLE patients show an increased level of IFN-β, especially in SLE patients with renal disease and in those with autoantibodies [104], suggesting the potential involvement of different types of IFN pathways in SLE pathogenesis.

Unlike other type I IFN family members, IFN-κ is selectively expressed in epidermal keratinocytes, and its levels significantly increase upon viral infection [105]. IFN-κ can trigger monocytes and DC to release cytokines and are also particularly effective in inhibiting inducible IL-12 release from monocytes [106]. Although few studies of IFN-κ have focused on SLE, genetic studies have shown that IFN-κ SNPs are associated with increased serum levels of type I IFN in SLE patients [107]. In SLE patients, increased skin production of IFN-κ by lupus keratinocytes drives the overproduction of IL-6 [108], suggesting a specific pathological role of IFN-κ in cutaneous lupus.

## 6. Type III IFNs in SLE

Although type III IFNs are thought to function as a pathogen defense by maintaining the mucosal immune system, it has also been suggested that type III IFNs are involved in the pathogenesis of SLE. Similar to type I IFNs, serum levels of IFN-λ1 are elevated in SLE patients and, more importantly, are positively correlated with lupus disease activity and anti-dsDNA levels, as well as being negatively correlated with complement level [109], perhaps partially due to an IFN-λ1-mediated increase in the level of Th17-derived cytokines [110] (Figure 3). Notably, elevated serum levels of IFN-λ3 are significantly correlated with higher SLE disease activity and decreased complement level, and genetically, the *IFNL3/4* SNP alleles that contain dysfunctional *IFNL4* in combination with low production of IFN-λ3 seem to be risk factors for lupus nephritis [111]. Persistently increased levels of IFN-λ have been suggested to be associated with unfavorable histological response in the treatment of lupus nephritis [112]. An SLE observational study found that IFN-λ1 is usually associated with milder disease, while high levels of type I IFNs tend to be associated with severe SLE with nephritis and arthritis [113]. Among the different type III IFNs, IFN-λ3 appears to be associated with the extent of lupus activity and, in particular, with active serosal and cutaneous disease, which may be triggered by anti-Ro/SSA antibodies [114]. Altogether, although the mechanism of the role of IFN-λs in SLE remains unclear, the evidence is accumulating for pathological adjuvant activity in the development and disease progression of SLE.

## 7. Targeting Type I IFN and IFN Signaling Pathways in SLE

Studies of murine lupus models and serial studies of SLE patients have shown that the IFN pathways are involved in a significant increase in the expression of IFN-regulated genes, the broad enhancement and regulation of immune cells, and autoimmune inflammation in SLE. Targeting IFNs and associated signaling molecules in IFN-mediated pathways has led to important achievements in the development of novel drugs for SLE. Presently, the main development in the search for anti-SLE drugs which target IFN pathways involves targeting type I IFNs, including employing monoclonal antibodies against IFN-α or its receptor IFNAR, or the use of a therapeutic vaccine to induce polyclonal anti-IFN-α neutralizing antibodies. Although therapeutic applications targeting type III IFNs have not yet been developed, trials investigating the use of small molecules to target JAK-STAT pathways are ongoing. IFN-targeting therapies are summarized in Table 1.

There are three therapeutic monoclonal antibodies, which directly inhibit IFN-α, namely rontalizumab, sifalimumab, and AGS-009. Rontalizumab is a humanized IgG1 monoclonal antibody, which neutralizes IFN-α and has an acceptable safety profile. Rontalizumab was found to cause a sustained decline in the expression of interferon regulated genes (IRGs) in a phase I dose-escalation study of mildly active SLE patients [122]. In the following phase II study (NCT00962832), 238 patients with moderate-to-severe active SLE were recruited and randomized to receive either rontalizumab or placebo [115]. The efficacy response rates between the two groups, which were assessed by the British Isles Lupus Assessment Group (BILAG) index (primary endpoint) and the SLE responder index response (SRI)-4 at week 24 (secondary endpoint), were found to be similar. Therefore, the development of rontalizumab has been discontinued due to failure to meet the primary endpoint. Sifalimumab is a fully human IgG1 monoclonal antibody, which neutralizes IFN-α. It showed both a satisfactory tolerability profile and efficacy profile in a phase I study [123]. In the following phase IIb trial (NCT01283139), 431 patients with moderate-to-severe active SLE were randomized to receive monthly intravenous sifalimumab at a dose of 200, 600, or 1200 mg, or placebo [116]. The results showed that a greater percentage of patients who received sifalimumab (all dosages) met the primary endpoint (percentage of patients achieving an SRI response at week 52) compared to the placebo group. Therefore, sifalimumab may be a promising treatment option for adults with SLE. AGS-009 is a humanized IgG4 monoclonal anti-IFN-α antibody, which has shown satisfactory tolerability profiles in a phase Ia single-dose-escalation study [117].

In addition to directly targeting IFN-α, anifrolumab, a fully human IgG1κ monoclonal antibody directed against IFNAR1, targets the type I IFN receptor. After a successful phase I and early phase II studies, a phase IIb double-blind placebo-controlled randomized control trial of 305 adults with moderate-to-severe SLE (MUSE trial, NCT01438489) showed positive results on the composite primary endpoint SRI-4 [118]. This trial also found that anifrolumab is especially effective for a high baseline IFN gene signature compared with a low baseline IFN gene signature and that anifrolumab showed promising results in patients with cutaneous and arthritic manifestations. Additionally, two phase III trials (TULIP 1 and TULIP 2, NCT02446899 and NCT02446912) assessed the efficacy and safety of anifrolumab in moderately-to-severely active autoantibody-positive SLE patients. In August 2018, the promoter announced that the TULIP1 Phase III trial did not reach its primary endpoint (SRI-4 at week 52); however, the final results are still pending.

Interferon-α-kinoid (IFN-K) is a therapeutic vaccine comprised of IFN-α2b with a carrier protein to induce the production of polyclonal anti-IFN-α neutralizing antibodies. In earlier animal studies, it was found that using IFN-K to immunize human IFN-α transgenic mice could induce the mice to produce antibodies that neutralize IFN-α in sera from SLE patients [124,125]. Moreover, in phase I/II randomized double-blind placebo-controlled dose-escalation study (NCT01058343), IFN-K was found to be well-tolerated and significantly reducing the expression of the IFN signature compared to placebo in 28 patients with mild-to-moderate SLE [119]. However, no differences were observed in disease activity scores (SLEDAI-2K and BILAG) nor were significant changes in baseline levels of C3, C4, or anti-dsDNA levels observed between the groups. Another phase IIb randomized double-blind placebo-controlled study is ongoing to evaluate the effect of IFN-α-kinoid on the neutralization of the IFN gene signature and its clinical efficacy in SLE patients (NCT02665364).

Other candidate targets transduced by type I and type III IFN signaling pathways are involved in JAK-STAT signaling pathways. Type I and type III IFNs signal through JAKs to induce IFN-stimulated gene expression; therefore, several JAK inhibitors used for the treatment of other autoimmune diseases are also under evaluation for their efficacy in treating SLE (Table 1). Additionally, following satisfactory results in phase II trials, two phase III randomized double-blind placebo-controlled parallel-group studies of the use of the JAK1/JAK2 inhibitor baricitinib in SLE patients are in progress (NCT03616964 and NCT03616912) [120].

So far, various agents, which target IFN-α receptors or type I IFN receptors, have failed to meet their primary endpoints in SLE clinical trials. Other than the drugs not being efficacious, several reasons can be proposed for these clinical trial failures, including the clinical heterogeneity of eligible patients, the outcome measurement not being suitable for the clinical trials, the measurement being unable to reflect changes accurately over time, site-investigator bias for the recruitment, or other errors in trial design [126,127]. For rontalizumab, although a phase II study showed safe and well-tolerated profiles in patients with active extrarenal lupus, it did not achieve its primary efficacy endpoint, probably due to a discrepancy in the numbers of SLE patient subgroups (high or low IFN signature metric (ISM)) leading to suboptimal drug efficacy [115]. In a sifalimumab trial, although it demonstrated significant clinical effects across several different validated endpoints and in organ-specific assessments of disease activity [116], further clinical trials were not performed due to the loss of sponsorship. In a phase III study of anifrolumab, TULIP 1 failed to reach its primary goal, with the proportion of patients achieving an SRI-4 at week 52 being similar in the anifrolumab and placebo groups [118]; meanwhile, the final results for TULIP 2 are still pending. It is conceivable that the IFN signatures in the recruited SLE patients restricted the efficacy of anifrolumab on the disease. However, more detailed patient recruitment with genetic background selection may improve trials of the efficacy of anifrolumab to treat SLE.

Other potential future SLE therapies may involve the inhibition of IFN receptor subunits, such as Tyk2. The inhibition of Tyk2 selective inhibitors (BMS-986165) was addressed in early clinical and preclinical investigations [128], and a phase II study is ongoing to characterize the long-term safety and tolerability of Tyk2 selective inhibitors in SLE (NCT03920267). Future SLE therapies may also target the inhibition of other IFN receptor subunits, such as IFNLR1 (IL-28Rα) and IL-10R2; however, to the best of the authors’ knowledge, no clinical trials are ongoing.

## 8. Concluding Remarks

Type I and type III IFNs were originally investigated for their antiviral activity; however, recent evidence has shown that they also possess an immunomodulatory function in autoimmunity. Type I and type III IFNs share several properties in common; however, there appear to be temporal and kinetic differences in the signaling pathways of the consequent immune pathologies. Despite these differences, both type I and type III IFNs are central mediators in the pathogenesis of SLE. Therefore, novel therapeutic strategies targeting IFNs and IFN signaling pathways are being developed, and these seem promising for modulating disease activity in SLE. In the future, the selection of the most effective drugs for SLE patients may be based on the expression of the IFN signature or an in-depth personalized characterization involving IFN signaling pathways.

## Figures and Tables

**Table 1:** Therapies targeting type I interferons (IFNs) and IFN signaling pathways in systemic lupus erythematosus (SLE).

Therapy	Mechanism of Action	Current Development Stage	Ref.
Rontalizumab	Humanized IgG1 mAb against IFN-α	Phase II, completed	[115]
Sifalimumab	Fully human IgG1 against IFN-α	Phase IIb, completed	[116]
AGS-009	Humanized IgG4 mAb against IFN-α	Phase I, completed	[117]
Anifrolumab	Fully human IgG1κ mAb against IFNAR1	Phase II (MUSE trial), completed Phase III (TULIP 1 and 2), completed	[118] NCT02446899 NCT02446912
IFN-α kinoid (IFN-K)	Therapeutic vaccine of IFN-α2b	Phase I/II, completed	[119]
Baricitinib	JAK1/JAK2 inhibitor	Phase II, completed Phase III, recruiting	[120] NCT03616964 NCT03616912
Tofacitinib	JAK1/JAK2/JAK3 inhibitor	Phase I, completed Phase I/II, recruiting	NCT02535689 NCT03288324
Ruxolitinib	JAK1/JAK2 inhibitor	Preclinical	[121]
CC-930	JAK1/JAK2/JAK3 inhibitor	Phase II, terminated (discoid lupus)	NCT01466725
GSK2586184	JAK1 inhibitor	Phase I, completed Phase II, terminated	NCT01687309 NCT01777256
